# The uptake of [^18^F]-fluorodeoxyglucose by the renal allograft correlates with the acute Banff scores of cortex inflammation but not with the 1-year graft outcomes

**DOI:** 10.3389/frtra.2023.1236751

**Published:** 2023-08-25

**Authors:** Hélène Fank, Laurent Weekers, Pierre Lovinfosse, Hans Pottel, Laurence Seidel, Alexandre Jadoul, Antoine Bouquegneau, Catherine Bonvoisin, Christophe Bovy, Stephanie Grosch, Pauline Erpicum, Roland Hustinx, François Jouret

**Affiliations:** ^1^ Division of Nephrology, Department of Internal Medicine, University of Liège Hospital (ULiège CHU), Liège, Belgium; ^2^ Division of Nuclear Medicine and Oncological Imaging, University of Liège Hospital (ULiège CHU), Liège, Belgium; ^3^ Department of Public Health and Primary Care, KU Leuven Campus Kulak Kortrijk (KULAK), Kortrijk, Belgium; ^4^ Department of Medico-Economic Information and Biostatistic, University of Liège Hospital (ULiège CHU), Liège, Belgium; ^5^ Division of Renal Pathology, Unilab, University of Liège Hospital (ULiège CHU), Liège, Belgium

**Keywords:** kidney transplantation, acute rejection, per cause biopsy, ^18^F-fluorodeoxyglucose, positron emission tomography, diagnosis, prognosis

## Abstract

**Introduction:**

[^18^F]FDG PET/CT noninvasively disproves acute kidney allograft rejection (AR) in kidney transplant recipients (KTRs) with suspected AR. However, the correlation of biopsy-based Banff vs. PET/CT-based scores of acute inflammation remains unknown, as does the prognostic performance of [^18^F]FDG PET/CT at one year *post* suspected AR.

**Methods:**

From 2012 to 2019, 114 [^18^F]FDG-PET/CTs were prospectively performed in 105 adult KTRs who underwent *per cause* transplant biopsies. Ordinal logistic regression assessed the correlation between the extent of histological inflammation and the mean standardized [^18^F]FDG uptake values (mSUV_mean_). Functional outcomes of kidney allografts were evaluated at one year post *per cause* biopsy and correlated to mSUVmean.

**Results:**

A significant correlation between mSUV_mean_ and acute Banff score was found, with an adjusted *R*^2^ of 0.25. The mSUV_mean_ was significantly different between subgroups of “total i”, with 2.30 ± 0.71 in score 3 vs. 1.68 ± 0.24 in score 0. Neither the function nor the survival of the graft at one year was statistically related to mSUV_mean_.

**Discussion:**

[^18^F]FDG-PET/CT may help noninvasively assess the severity of kidney allograft inflammation in KTRs with suspected AR, but it does not predict graft outcomes at one year.

## Introduction

Kidney transplantation (KTx) represents the best option for patients with end-stage renal disease (ESRD). Acute kidney allograft rejection (AR) is one of the main causes of allograft loss (1). The gold standard for the diagnosis of AR currently relies on renal transplant biopsy (2). The systematic histological analysis of a renal sample allows for the identification of both type and degree of rejection according to the conventional Banff classification, which provides a standardized scoring of kidney injury (3). More specifically, the Banff classification semi-quantifies both acute and chronic inflammation in five histological compartments, either individually or collectively: glomeruli, tubules, interstitium, peritubular capillaries, and arteries (3).

Although an ultrasound-guided core needle biopsy of kidney allograft is regarded as relatively safe, it remains an invasive procedure with a ∼7% rate of complications (4) including bleeding or development of an arteriovenous fistula. Furthermore, inter-observer variability and sampling errors limit its benefits (5). Therefore, the development and validation of noninvasive methods to detect T cell-mediated rejection (TCMR) and antibody-mediated rejection (ABMR) would be highly valuable. Urinary and plasma biomarkers of acute and chronic renal rejections have been identified and are currently under clinical validation (6–8). Imaging techniques have significantly improved over the past decades, thereby proposing another approach to the noninvasive diagnosis of AR. More specifically, ^18^F-fluorodeoxyglucose [(^18^F)FDG] positron-emission tomography combined with computed tomography (PET/CT) as first-line examination with a high negative predictive value may help noninvasively prevent avoidable transplant biopsies in kidney transplant recipients (KTRs) with clinically suspected AR but no histological lesions (9, 10). Indeed, the AR-associated immunological reaction against donor antigens induces the recruitment of mononuclear leukocytes into the renal transplant, which corresponds to the core of the Banff classification. The boosted metabolism of these inflammatory cells can be assessed by PET quantification of the renal uptake of the radiotracer, [^18^F]FDG. The lack of detectable signals, therefore, suggests the absence of active inflammation.

In the present *post hoc* analysis of our prospective studies, we test the correlation of biopsy-based Banff vs. PET-based scores of acute inflammation in renal transplants. Furthermore, we assess the prognostic performance of the renal uptake of [^18^F]FDG regarding allograft outcomes at one year post *per cause* biopsy.

## Patients and methods

### Patients

The study was approved by the Institutional Review Board of the University of Liege: #B707201215598. Between December 2012 and December 2019, we prospectively enrolled adult KTRs undergoing a *per cause* transplant biopsy for suspected AR (i.e., an increase in serum creatinine levels >30% of the baseline value or delayed graft function) (11). Signed informed consent was obtained from all patients. These cohorts have been previously described, as “training” (9) and “validation” (10) sets.

### Histopathology

Biopsies were assessed by two pathologists (SG and ChB), who were blinded to the results of [^18^F]FDG-PET/CT imaging. Banff 2017 classification [in gold-standard practice at the time of the (^18^F)-FDG PET/CT procedures] was conventionally used (12). The threshold retained to define borderline rejection was i0t1. Histological lesions were scored as continuous variables (from zero to three) on the basis of the leukocyte infiltration severity in each component: glomeruli (g); peritubular capillaries (ptc); arteries (v); tubules (t); and interstitium (i). The acute Banff score was conventionally defined as the sum (from 0 to 15) of g, ptc, v, t, and i. The Banff “total i” score (from 0 to 3) corresponding to the total cortical inflammation, including scarred and non-scarred cortex, was also examined. The IFTA score (from 0 to 3) corresponds to interstitial fibrosis and tubular atrophy in the cortex. All biopsies were immunostained for polyoma BK virus.

### [^18^F]FDG-PET/CT imaging

The PET/CT procedure was performed using cross-calibrated Philips GEMINI TF Big Bore or TF 16 PET/CT systems (Philips Medical Systems, Cleveland, OH, USA) at 194 ± 19 min after intravenous administration of a mean dose of 243 ± 35 MBq of ^18^FDG before any modification of immunosuppressive regimens, as previously described (9, 10). A low-dose helical CT (5-mm slice thickness, 120-kV tube voltage, and 40-mAs tube current-time product) centered on the renal transplant was performed, followed by a PET emission scanning with two-bed positions each lasting 4 min. Images were reconstructed using iterative list mode time-of-flight algorithms, and corrections for attenuation, dead-time, random, and scatter events were applied. The PET/CT procedure was performed within a 48-h period of the ultrasound-guided renal transplant biopsy. All [^18^F]FDG-PET/CTs were acquired in fasting conditions and without the administration of contrast agents or diuretics. PET/CT images were read independently by two experienced nuclear medicine physicians (PL and AJ), who were blinded to the histological results. The mean glycemia at the time of tracer injection was 109 ± 27 mg/dl. Conventionally, the glycemia at the time of [^18^F]FDG-PET/CT needed to be lower than 200 mg/dl, as recommended by the EANM (European Association of Nuclear Medicine and Molecular Imaging) for PET/CT in diabetic patients. Four volumes of interest (VOIs) of 1 ml were manually drawn in the cortical regions of both the upper (*n* = 2) and lower (*n* = 2) poles of the renal transplant at a distance from the pelvicalyceal zone. The SUV_mean_ was measured in each VOI, with no threshold activity, and the mean of these four SUV_mean_ was calculated (mSUV_mean_).

### Statistics

The data are presented as the mean ± standard deviation (SD) or as the median and interquartile range [IQR] for quantitative variables. Frequencies of qualitative variables are presented as numbers. Groups were compared using one-way analysis of variance (ANOVA) followed by *post hoc* Scheffé test or Kruskal–Wallis tests for quantitative variables, and the *χ*^2^ test (or Fisher exact test) was used for the qualitative variables. The association between the extent of Banff-scored histological inflammation and mSUV_mean_ was assessed by the ordinal logistic regression. The Spearman correlation was calculated between the mSUV_mean_ and the acute Banff score and the g + ptc score. In the second part of the study focusing on the outcomes of kidney allografts according to mSUV_mean_ or Banff-based histological parameters (i.e., “total i” score and acute Banff score), patients who underwent a second *per cause* biopsy during the one-year follow-up were excluded. The mSUV_mean_ was distributed in tertiles. The “total i” score was used with a threshold of 25% as suggested by Mengel et al. (13). The acute Banff score was considered continuous variables. The 1-year survival of the renal graft, defined as no graft loss after the *per cause* biopsy/PET/CT was analyzed by univariate logistic regression according to mSUV_mean._, total i, and acute Banff score. The graft overall survival was analyzed by the univariate Cox regression according to mSUV_mean._, total i, and acute Banff score model and represented by the Kaplan–Meier curves. Changes since baseline in serum creatinine and eGFR levels were assessed at 12 ± 1 months after the *per cause* biopsy/PET/CT by the Wilcoxon sign rank test. Results were considered significant at the 5% confidence level (*p* < 0.05). Calculations were done in SAS version 9.4 and figures in R version 4.2.2.

## Results

### Clinical and histological features of the cohort

**Figure 1:**
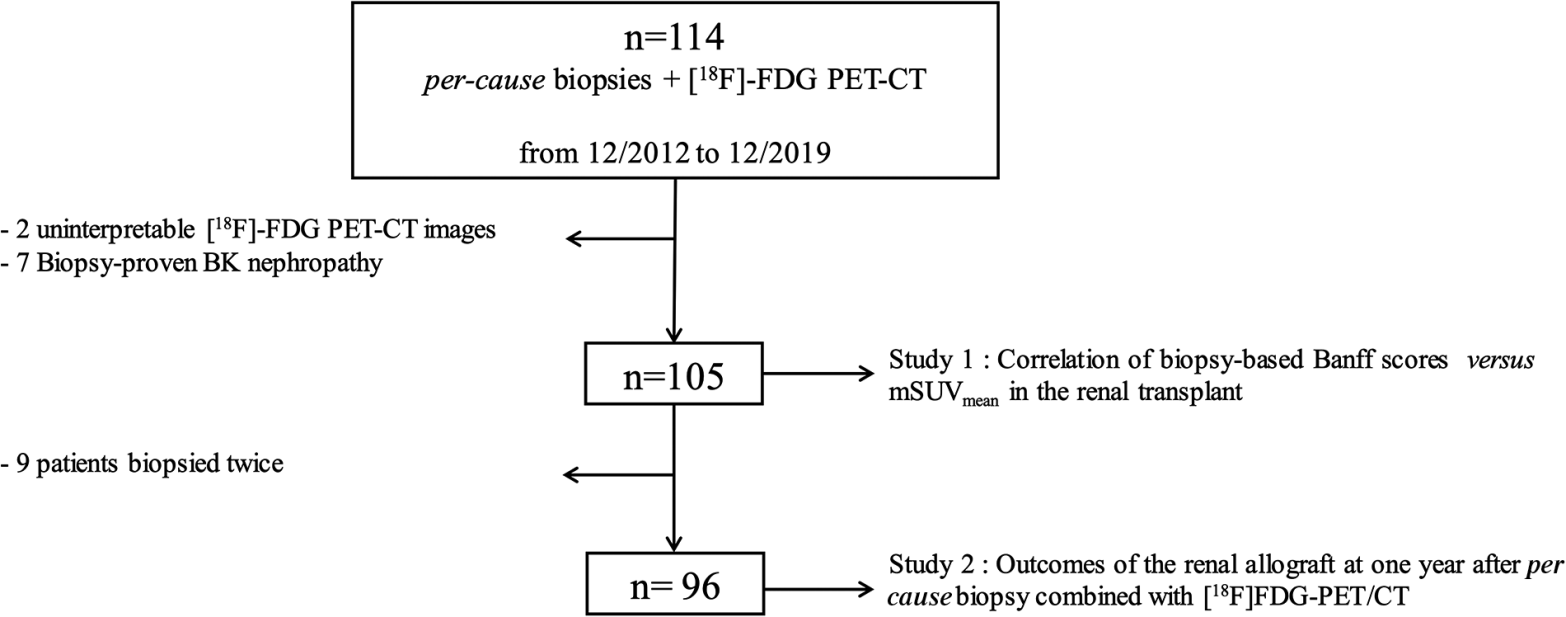
Flowchart of the cohort.

Between December 2012 and December 2019, 114 [^18^F]FDG-PET/CTs were performed prospectively in 105 KTR with suspected AR. The indication of the allograft biopsy was part of the medical management at the discretion of the physician in charge. Histology and imaging were independently analyzed. Biopsy-proven polyoma-BK nephropathies (*n* = 7) and uninterpretable PET/CT images (*n* = 2) were excluded (Figure 1). The clinical and biological characteristics of the cohort are summarized in Table 1. The mean age of the cohort at the time of biopsy was 47 ± 14 years, with an M/F ratio of 1.5.

**Table 1:** Clinical and biological characteristics of the cohort.

	Cohort (*n* = 105)	Normal (*n* = 62)	Borderline (*n* = 19)	Acute rejection (*n* = 21)	Other (*n* = 3)	*p*-value
Recipients						
Age (year): m ± SD	47 ± 14	48 ± 14	44 ± 17	47 ± 13	44 ± 11	0.81
Sex (Male/Female): *n*	63/42	38/24	10/9	13/8	2/1	0.92
BMI (Kg/m^2^): m ± SD	26 ± 5	26 ± 5	26 ± 5	26 ± 5	20 ± 1	0.15
Donors						
Age (year): m ± SD	42 ± 13	42 ± 12	42 ± 15	44 ± 15	32 ± 16	0.57
Sex (Male/Female): *n*	50/54	30/32	7/11	11/10	2/1	0.75
Donor type (DBD/DCD/LD): *n*	74/20/10	44/14/4	13/2/3	14/4/3	3/0/0	0.68
Transplantation						
Rank (1st/2nd/3rd): *n*	92/11/1	54/7/1	16/2/0	19/2/0	3/0/0	1.0
CIT (min): m ± SD	625 ± 306	641 ± 282	639 ± 375	558 ± 319	681 ± 323	0.73
HLA mismatches: m ± SD						
Locus A	0.9 ± 0.6	0.9 ± 0.5	0.9 ± 0.6	1.0 ± 0.7	1.0 ± 0.0	0.95
Locus B	1.2 ± 0.5	1.0 ± 0.5	1.3 ± 0.6	1.2 ± 0.5	1.3 ± 0.6	0.20
Locus DR	0.8 ± 0.6	0.8 ± 0.6	0.7 ± 0.5	0.9 ± 0.6	1.3 ± 0.6	0.25
Early graft function (immediate/slow/delayed): *n*	66/27/11	37/19/6	12/2/4	15/6/0	2/0/1	0.12
Status at the time of biopsy						
Maintenance immunosuppression: *n*						
CNI (CsA/FK/none)	13/89/3	7/54/1	4/14/1	2/18/1	0/3/0	0.58
Antimetabolite (MMF/MPA/AZA/none)	77/19/2/7	48/9/1/4	14/3/1/1	14/5/0/2	1/2/0/0	0.45
mTOR inhibitor (yes/no)	5/100	3/59	1/18	1/20	0/3	1.00
CS (yes/no)	84/21	49/13	16/3	16/5	3/0	0.92
Duration of KTx at biopsy (d): M [P25; P75]	272 (32–1,721)	99 (28–1,316)	1,191 (64–2,117)	649 (178–1,366)	1,150 (6–3,158)	0.32
Creatinine (mg/dl): M [P25; P75]	1.95 (1.62–2.67)	1.85 (1.60–2.53)	2.07 (1.59–2.54)	2.3 (1.71–2.92)	1.87 (1.63–5.53)	0.40
eGFR (ml/min/1.73 m^2^): m ± SD	34.5 ± 15.0	36.6 ± 15.4	33.3 ± 15.3	29.6 ± 12.5	31.5 ± 20.5	0.29
DSA (none/class I alone/class II alone/class I + II): *n*	79/8/11/4	52/3/4/1	14/1/2/1	12/4/3/2	1/0/2/0	0.027

Data are expressed as mean (m) ± standard deviation (SD) and as median (M) ± [P25; P75].

AZA, azathioprine; BMI, body mass index; CIT, cold ischemic time; CNI, calcineurin inhibitors; CS, corticosteroids; CsA, cyclosporin A; DBD, donor after brain death; DCD, donor after circulatory death; DSA, donor-specific antibodies; eGFR, estimated glomerular filtration rate; FK, tacrolimus; KTx, kidney transplantation; LD, living donor; MMF, mycophenolate mofetil; MPA mycophenolic acid; mTOR, mammalian target of rapamycin.

Biopsies were performed after a median time of 272 [32; 1,721] days *post*-transplantation. At the time of biopsy, the mean eGFR was 34.4 ± 15.0 ml/min/1.73 m^2^. The prevalence of biopsy-proven AR and borderline was 20.0% and 18.1%, respectively. AR was acute TCMR in 14 cases, whereas ABMR or mixed cases were found in 3 and 4 cases, respectively. The cause of graft failure in the “other” group included acute tubulointerstitial nephropathy and non-active chronic rejection. No difference was observed between groups regarding the clinical and biological characteristics of kidney donors and recipients (Table 1). At the time of the biopsy, a statistically significant difference was found in terms of the presence of donor-specific antibodies (DSA), which were more commonly observed in the “AR” vs. “other” groups. Serum levels of creatinine were similar in all groups.

### Correlation of biopsy-based Banff scores vs. mSUV_mean_ in the renal transplant

The mSUV_mean_ of the 105-case cohort was 1.82 ± 0.44. We observed a significant difference in the mSUV_mean_ among groups (*p *< 0.0001). More specifically, the AR group was characterized by a significantly higher value of mSUV_mean_ (2.28 ± 0.57) compared to the “normal” (1.67 ± 0.22) and “borderline” (1.87 ± 0.46) groups. The mSUV_mean_ in the “other” group was 1.45 ± 0.24. Of note, the mSUV_mean_ of the seven patients with biopsy-proven polyoma-BK nephropathies was 2.5. The distribution of the “total i” score was 0 (58.1%), 1 (19.1%), 2 (9.5%), and 3 (13.3%). The distribution of the “IFTA” score was 0 (60.0%), 1 (30.5%), 2 (3.8%), and 3 (5.7%). The highest value of the acute Banff score was 12, while 54.2% of biopsies were scored as 0.

**Figure 2:**
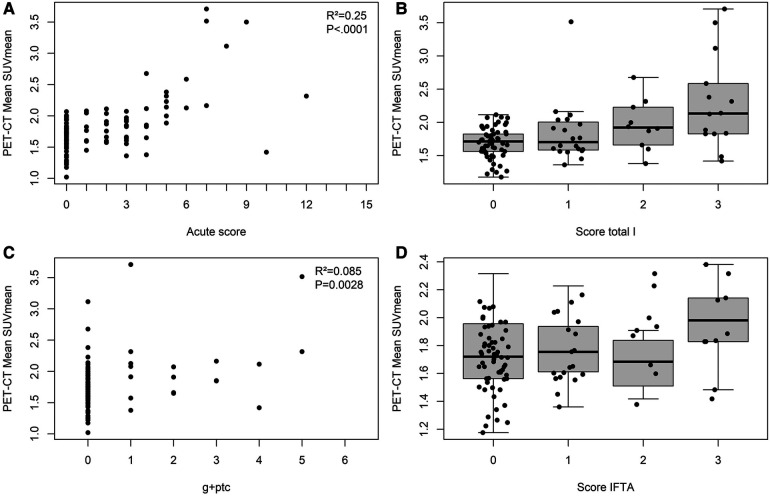
Correlations of biopsy-based Banff vs. PET/CT-based scores in the renal transplant. Panel **A**: Positive correlation between mSUVmean and acute composite Banff score (*R*^2 ^= 0.25, *p *< 0.0001). Panel **B**: Boxplot showing mean values of SUVmean in histopathological categories of total i score; 0 *n* = 61), 1 (*n* = 20), 2 (*n* = 10), and 3 (*n* = 14). The mSUVmean reached 1.68 ± 0.24, 1.84 ± 0.46, 1.96 ± 0.38, and 2.30 ± 0.71 for the score total i of 0, 1, 2, and 3, respectively. Ordinal logistic regression demonstrated a significant difference in mSUVmean among groups (*p *< 0.0001). Panel **C**: Positive correlation between mSUVmean and g + ptc (*R*^2^ = 0.085, *p *= 0.0028). Panel **D**: Boxplot showing mean values of SUVmean in histopathological categories of IFTA score; 0 (*n* = 63), 1 (*n* = 32), 2 (*n* = 4), and 3 (*n* = 6). The mSUVmean reached 1.83 ± 0.50, 1.80 ± 0.35, 1.67 ± 0.21, and 1.97 ± 0.32 for the score IFTA 0, 1, 2, and 3, respectively. Ordinal logistic regression did not show any significant difference in mSUVmean among groups (*p* = 0.91).

A significant positive correlation between mSUV_mean_ and the acute composite Banff histological score was calculated, with an *R*^2^ of 0.38 (*p *< 0.0001) (Figure 2A). Increasing grades (from ti0 to ti3) of leukocyte infiltration in total allograft cortex were significantly associated with increasing mSUV_mean_ (*p* < 0.0001), with 2.30 ± 0.71 in score 3 vs. 1.68 ± 0.24 in score 0 (Figure 2B). Focusing on the microvascular inflammation-related parameters, we observed a positive and significant correlation between mSUV_mean_ and g + ptc scores, with an *R*^2^ of 0.085 (*p *= 0.0028) (Figure 2C). Conversely, we did not detect any significant difference in mSUV_mean_ among IFTA groups (*p* = 0.91) (Figure 2D).

### Outcomes of the renal allograft at one year after *per cause* biopsy combined with [^18^F]FDG-PET/CT PET/CT

**Figure 3:**
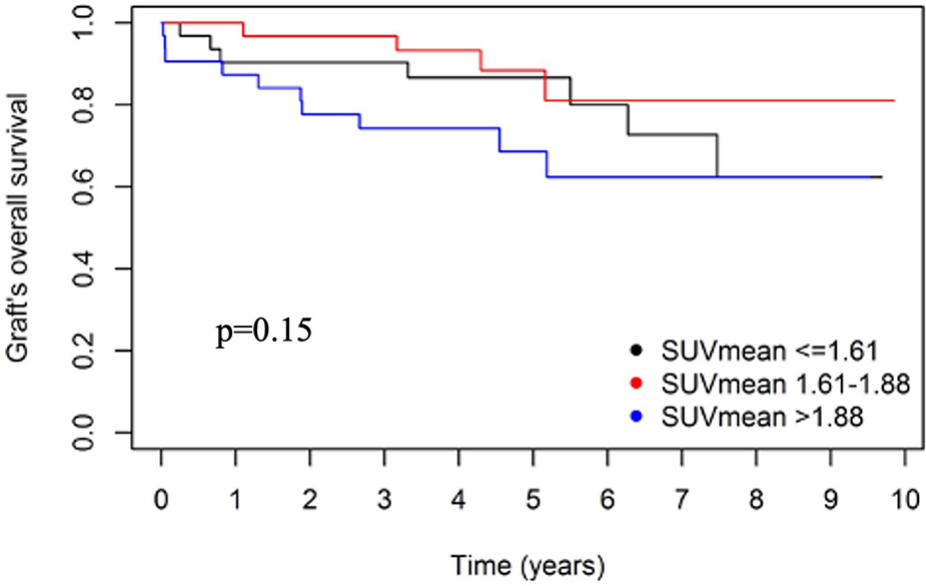
Overall graft survival according to tertile of mSUV_mean_. Kaplan–Meier curve for the mSUVmean. Tertiles of mSUV_mean_ do not show a prognostic significance (*p *= 0.15).

Survival analysis was calculated using only the first *per cause* biopsy of each patient. Accordingly, nine patients were excluded (Figure 1). The histological findings of the first biopsy of these nine patients showed normal histology in five patients and AR in four patients. The median time between the first and second biopsies was 214.5 (21; 1,559) days. The remaining 96 patients were followed-up until 29 November 2022. At one year *post per cause* biopsy, serum creatinine (SCr) levels dropped significantly from 1.87 (1.6–2.5) mg/dl to 1.70 (1.3–2.2) mg/dl (*p* = 0.039). Among the 93 patients still alive at one year *post per cause* biopsy, seven (7.5%) lost their graft: five patients lost their grafts because of rejection, one because of recurrence of the initial disease, and one for an undefined cause. The graft loss at one year *post per cause* biopsy was not related to mSUV_mean_ or “total i” score (Table 2). A non-significant trend was observed for the acute Banff score (OR = 1.24, *p* = 0.064). No significant association was found between the change in SCr levels and mSUV_mean_ (or the histological parameters) at one year *post per cause* biopsy (Table 3). The overall graft survival was not influenced by the mSUV_mean_ (*p* = 0.15) or the “total i” score (*p* = 0.26) at the time of the first *per cause* allograft biopsy (*p* = 0.15) (Figure 3). Still, the risk of graft loss was significantly increased according to the acute Banff score (OR = 1.17, *p* = 0.027).

**Table 2:** Comparison of patients with and without graft loss according to imaging and histological parameters at one year.

Variable	Categories	Functional graft at 1 year (*n* = 86)	Graft lost at 1 year (*n* = 7)	OR	*p*-value
mSUV_mean_ (Tertile)	≤1.61	28 (90.3)	3 (9.7)	1.0	0.32
	(1.61; 1.88)	31 (100.0)	0 (0.0)	0.13	
	>1.88	27 (87.1)	4 (12.9)	1.3	
Total i score	0–1 (<25%)	69 (94.5)	4 (5.5)	1.0	0.17
	2–3 (>25%)	17 (85.0)	3 (15.0)	3.0	
Acute score (continuous variable)		1.68 ± 2.3	3.71 ± 4.9	1.2	0.064

Data are expressed as mean (m) ± standard deviation (SD) and as number and frequencies (%); univariate logistic regression model.

**Table 3:** Evolution of renal parameters (serum creatinine levels and eGFR) according to imaging and histological parameters at one year.

	Delta Creatinine 1Y (mg/dl) M [P25; P75]	*p*-value	Delta eGFR (MDRD) 1Y (ml/min/1.73 m^2^) m ± SD	*p*-value
mSUVmean				
mSUV_mean _≤ 1.61	0.05 (−0.14; 0.42)	0.43	−4.3 ± 12.2	0.65
mSUV_mean_ (1.61; 1.88)	0.20 (0.00; 0.66)		−7.4 ± 17.0	
mSUV_mean _> 1.88	−0.08 (−1.05; 0.71)		−4.0 ± 19.3	
Total i				
Total i 0–1 (<25%)	0.06 (−0.14; 0.56)	0.81	−4.6 ± 14.3	0.95
Total I 2–3 (>25%)	0.28 (−1.11; 0.28)		−7.4 ± 22.7	

Data are expressed as mean (m) ± standard deviation (SD) and as median (**M**) ± [P25; P75].

## Discussion

Various non-invasive tools are under investigation to rule out the diagnosis of AR in order to mitigate the risks of systematic use of renal allograft biopsies. More specifically, we and others have suggested that imaging-based approaches may be helpful in both structural and functional assessments of the entire renal allograft, which contrasts with the biopsy-based information limited to small-size samples. [^18^F]FDG-PET/CT is routinely used for the characterization, staging, and follow-up of inflammatory processes of various origins (14). The usefulness of [^18^F]FDG-PET/CT in the management of KTRs presenting with suspected AR has been demonstrated in both training and validation cohorts (9, 10). In our first pilot study published in 2015, sensitivity and specificity of [^18^F]-FDG PET/CT in diagnosing acute rejection were 100% and 50% respectively, with a meanSUV threshold of 1.6. In this 2015 cohort, characterized by a 25% prevalence of biopsy-proven AR, the corresponding negative and positive predictive values were 100% and 43.75%, respectively. The proposed threshold of 1.6 of mSUV_mean_ was then validated in a second study of 86 patients. The ROC area under the curve was 0.86. Test sensitivity and specificity corresponding to the threshold value of 1.6 were 100% and 30%, respectively. In the present post-hoc analysis of these two prospective studies including 105 patients who have undergone a *per cause* biopsy, we show a positive and significant correlation between mSUV_mean_ and the acute Banff score. The ^18^F-FDG uptake by the renal graft also correlates with inflammation at the microvascular level. Moreover, increasing grades of “total i” scores were associated with increasing mSUV_mean_. These observations suggest that the renal uptake of the radiolabeled glucose analog [^18^F]FDG is correlated with the severity of biopsy-proven inflammation in the renal allograft. By contrast, the extent of fibrosis and tubular atrophy does not affect the renal [^18^F]FDG uptake. Of methodological note, similar results were found when focusing on 96 patients after excluding the eventual second biopsy. The radiotracer [^18^F]FDG follows a tissular distribution and cellular metabolism comparable to the glucose: it enters into cells via the glucose transporters (GLUT1), where it is phosphorylated by the hexokinase into [^18^F]FDG-6-phosphate and trapped in the cells. Hence, the intracellular accumulation of [^18^F]FDG-6-phosphate reveals a high cellular metabolism and is detectable by PET/CT (5, 15). Stimulated inflammatory cells are metabolically activated and show an increased expression of glucose transporters, which increases the uptake and accumulation of [^18^F]FDG in tissue sites of inflammation (14, 16). Our correlation observed between mSUV_mean_ and increasing grades of the “total i” score may thus reflect the accumulation of leucocytes metabolically active in the renal cortex, notably in the case of AR. Conversely, fibrotic tissue and atrophic tubules are less metabolically active, with no detectable impact on [^18^F]FDG uptake.

Historically, the lesions selected for scoring AR were the infiltration of the interstitium (i) and the tubules (t) by mononuclear cells, which are regarded as representative of the severity of TCMR (13). The “ti character” was added in Banff 2017 and is defined as inflammation in the total parenchyma, including scarred and non-scarred cortex, whereas the “i score” is limited to the non-scarred cortex (3). Compared with “i and t scores”, the “ti score” better reflects the molecular phenotypes of the tissue and is a better predictor of graft survival outcomes than the “i score” in cases where at least mild IFTA is present (13, 17). The correlation between [^18^F]FDG uptake and the severity of the histological lesions has been previously shown in a rat model of kidney AR. Reuter et al. (18) showed the most intense [^18^F]FDG signaling in animals with biopsy-proven AR characterized by large infiltrates of inflammatory mononuclear cells in the renal cortex (which mimics the “ti” score).

Although [^18^F]FDG uptake significantly correlates with the degree of histological inflammation, we could not demonstrate that mSUV_mean_ predicts the 1-year outcomes of kidney allografts. This dichotomy could be explained by the small number of cases in the different groups and the inherent low statistical power limiting the interpretation of these results. Another limitation of this study is the wide range of time between the transplantation and the date of the *per cause* biopsy [i.e., 272 (32; 1,721) days]. In the present cohort, the histological score “total i” does not predict the outcome of the graft, in contrast with the Mengel study including 104 patients with *per cause* biopsy (13). Note that “an event” in Mengel et al. was defined as (i) either graft loss or (ii) persistent (at least 3 months) eGFR <30 ml/min estimated by the Cockcroft–Gault formula. Prognostic studies currently performed on non-invasive urinary markers are also limited. Rabant et al. (19, 20) have shown that the CXCL10: Cr ratio, measured at the time of a *per cause* biopsy showing ABMR, can identify patients at high risk for kidney allograft loss. They also showed that CXCL9 and particularly CXCL10 urinary levels quantified at early *post*-transplantation might predict immunologic quiescence in clinically and histologically stable kidney recipients. Another study demonstrated that high urinary CXCL10 was associated with inferior endpoint-free graft survival and it was an independent predictor of long-term renal allograft outcomes, irrespective of histology results (21). Still, Hirt-Minkowski et al. could not demonstrate that urine CXCL10 monitoring had a beneficial effect on 1-year outcomes in a cohort of patients with protocol biopsy (22). However, the experimental designs of these studies, with repeated biomarker assessments and/or highly selected patients, are very different from the present study.

While [^18^F]FDG-PET/CT seems to be a promising non-invasive tool to entirely image the renal allograft, there are some limitations to its use: (i) the poor specificity of [^18^F]FDG tracer which accumulates in other inflammatory conditions, like tumors or infections; (ii) the restricted availability of PET/CT machine and its cost; (iii) the minor exposure to radiations originating from both PET and CT procedures; (iv) the 3-hour delay between [^18^F]FDG i.v. injection and image acquisition (10, 14). Of note, the current development of digital PET/CT would probably improve the image quality, while dropping the financial burden and reducing the radiation dose for the PET and CT portions (23, 24).

The interpretation of our *post hoc* analysis is limited by the small number of patients with biopsy-proven AR and the single-center nature of the study. Moreover, by purpose, the design of the present study does not take into account the final histological diagnosis but it focuses on the degree of inflammation. Due to the evolution of the Banff classification, the final diagnosis may change over time. Recent works suggest that there is a growing interest to automate the Banff classification combining histological features and omics to improve diagnosis and enable better risk stratification and therefore patient management (25).

The present work suggests an interesting approach to reducing the number of “unnecessary” biopsies (26). Additional prospective studies are needed to evaluate the potential interest of PET/CT in clinical decision-making and practical use, e.g., for monitoring treatment and adjusting the immunosuppressive regimen *post*-AR, as well as the combination of noninvasive imaging with the new urinary and blood biomarkers (like CXCL10 and CXL9). Moreover, it would be interesting to implement the mSUV_mean_ in web-based tools designed to classify acute kidney injury in KTRs (27).

## Conclusion

[^18^F]FDG-PET/CT may help to noninvasively assess the degree of histological inflammation in KTR with suspected AR and clinical indication of kidney allograft biopsy. The prognostic performance of mSUV_mean_ at 1 year could not be formally demonstrated in the present cohort.

## Data Availability

The original contributions presented in the study are included in the article/Supplementary Material, further inquiries can be directed to the corresponding authors.
